# Cross-Presentation of Synthetic Long Peptides by Human Dendritic Cells: A Process Dependent on ERAD Component p97/VCP but Not sec61 and/or Derlin-1

**DOI:** 10.1371/journal.pone.0089897

**Published:** 2014-02-27

**Authors:** Jérémie Ménager, Frédéric Ebstein, Romain Oger, Philippe Hulin, Steven Nedellec, Eric Duverger, Andrea Lehmann, Peter-Michael Kloetzel, Francine Jotereau, Yannick Guilloux

**Affiliations:** 1 INSERM U892, Nantes, France; 2 Université de Nantes, Nantes, France; 3 CNRS, UMR 6299, Nantes, France; 4 Institut of Biochemistry, Charité University Hospital, Humboldt University, Berlin, Germany; 5 Glycobiochimie, ICOA, Université d’Orléans, Orléans, France; University Paris Sud, France

## Abstract

Antitumor vaccination using synthetic long peptides (SLP) is an additional therapeutic strategy currently under development. It aims to activate tumor-specific CD8^+^ CTL by professional APCs such as DCs. DCs can activate T lymphocytes by MHC class I presentation of exogenous antigens - a process referred to as “cross-presentation”. Until recently, the intracellular mechanisms involved in cross-presentation of soluble antigens have been unclear. Here, we characterize the cross-presentation pathway of SLP Melan-A_16–40_ containing the HLA-A2-restricted epitope_26–35_ (A27L) in human DCs. Using confocal microscopy and specific inhibitors, we show that SLP_16–40_ is rapidly taken up by DC and follows a classical TAP- and proteasome-dependent cross-presentation pathway. Our data support a role for the ER-associated degradation machinery (ERAD)-related protein p97/VCP in the transport of SLP_16–40_ from early endosomes to the cytoplasm but formally exclude both sec61 and Derlin-1 as possible retro-translocation channels for cross-presentation. In addition, we show that generation of the Melan-A_26–35_ peptide from the SLP_16–40_ was absolutely not influenced by the proteasome subunit composition in DC. Altogether, our findings propose a model for cross-presentation of SLP which tends to enlarge the repertoire of potential candidates for retro-translocation of exogenous antigens to the cytosol.

## Introduction

The notion that the immune system can recognize and mount a response against tumors was initially postulated by Coley [1]. The development of immune responses against tumors *in vivo* involves the presentation of target structures on the cell surface of cancer cells, namely, tumor-associated antigens (TAA). These molecules must be presented effectively to effector cells of the immune system (NK, LT CD8^+^, CD4^+^,…) for the establishment of a lasting and beneficial immune response. Although anti-tumor immunity requires both innate and adaptive immune responses, it is generally accepted that CD8^+^ CTL are the most effective antitumor effector cells [2], [3].

The adaptive immune response depending on CTL involves TAA expression by tumor cells and requires TAA presentation by professional APCs. Among professional APC, DCs possess the unique ability, via co-stimulatory signals, to activate naive T lymphocytes in secondary lymphoid organs [4], [5]. Indeed, DCs take up extracellular TAA, process them intracellularly into antigenic peptides and load them onto major histocompatibility class I and class II molecules (MHC). The process whereby an exogenous antigen is acquired, processed and presented as peptide bound on MHC class I is known as “cross-presentation” [6], [7], [8]. Many compartments are involved in cross-presentation of soluble antigens [9] that critically depend on the nature of the antigen. Early endosomes internalize soluble antigens [10], the ER and Golgi apparatus are involved in presentation of endogenous antigens but can participate in the processing of antigens internalized by DCs [7], [11]. Furthermore, lysosomes have been recently reported to participate in cross-presentation of some antigens [12].

Current immunotherapy strategies are designed to provide either passive or active immunity against malignancies by harnessing the immune system to target tumors [13]. Among the various therapies, one common approach is therapeutic vaccination consisting in the injection of antigen from cancer cells in order to stimulate specific anti-tumor immunity. Many immunotherapy strategies, in particular those involving DCs, are under development [4], [14]. However, all of these strategies of therapeutic vaccination have so far exhibited low clinical benefit for patients [15].

A new immunotherapy strategy has emerged based on synthetic long peptides (SLP). SLP are usually 25–50 amino acids long and contain antigenic epitopes that require endocytosis and processing by professional APC such as DCs [15], [16]. Indeed, SLPs cannot be loaded exogenously on MHC-I and their processing requires a cross-presentation mechanism restricted to DCs for induction of a CTL response, thereby inducing anti-tumor immunity rather than tolerance [4], [17]. Several studies and clinical trials have been performed using SLP as vaccines with promising results against vulvar intraepithelial neoplasia lesions, cervical cancer and ovarian cancer [18], [19], [20], [21].

In this study, we designed a SLP from the Melan-A/MART-1 TAA. This SLP of 25 amino acids covers positions 16 to 40 of Melan-A/MART-1 (SLP_16–40_) and includes the A27L modification which allows a better anchoring of the immunodominant Melan-A/MART-1 26–35 epitope to the HLA-A*0201 molecule [22]. This SLP_16–40_ includes epitopes recognized by HLA class I restricted T-cell clones [23], [24]. A previous study, published by our group had shown that this SLP_16–40_ and its natural homologue are efficiently and durably cross-presented by DCs [25]. In addition our group has shown that cross-presentation of modified SLP_16–40_ results in efficient priming of a CD8 tumor reactive T cell repertoire. Nonetheless, to the best of our knowledge, the cellular mechanism involved in this cross-presentation by human DCs remains to be elucidated. Here, we characterize the cross-presentation pathway of SLP_16–40_. We show that it is dependent on early endosomes, followed by the ERAD pathway: retro-translocation into the cytosol and poly-ubiquitinylation of the SLP for proteasome degradation. Altogether, our results define the processing mechanism of SLP_16–40_ by DCs.

## Materials and Methods

### Culture Medium

Culture medium RPMI 1640 (Gibco BLR, Gaithersburg, MD) was supplemented with penicillin-streptomycin (100 U/ml and 100 µg/ml respectively; Life Technologies) and L-Glutamine (2 mM) (Life Technologies, Cergy-Pontoise, France) and either with 1% human plasma, 8% pooled human serum (pHS) or 10% fetal calf serum (FCS, Eurobio, Les Ulis, France).

### Cell Culture

HLA-A2 Monocytes were purified using centrifugal counter-flow elutriation (Clinical Transfer Facility CICBT0503, Dr. M. Grégoire, Nantes) and cultured for 4.5 days in RPMI 1640 2% human albumin in the presence of 1000 U/mL GM-CSF (Cellgenix) and 200 U/mL IL-4 (Cellgenix): MoDCs medium [26].

Maturation of Mo-DCs was induce by addition of 1000 U/mL TNFα (Cellgenix) and 50 µg/mL poly I:C (Sigma) to the culture (Maturation MoDCs medium). Each preparation of DC was checked for purity and differentiation by flow cytometry using the markers indicated below.

Human DC phenotype was determined by the expression of CD14, CD40, CD80, CD83 and HLA-DR (data not shown).

In cross presentation assays 10^6^ DCs were plated per well in 24 -well plates- pretreated with 3% polyHema (Sigma) for 16 hr to facilitate the harvest of DCs after the antigenic pulse.

The 10C10 clone was amplified as previously described [27].

### Synthetic Peptides

Synthetic long peptide Melan-A/MART-1_16–40_ (GHGHSYTTAEELAGIGILTVILGVL) (SLP_16–40_) and synthetic short peptide Melan-A/MART-1_26–35_ with the A27L modification (ELAGIGILTV), were synthesized with purity greater than 95% and purchased from Millegen (Labege, France). Synthetic fluorescent long peptides Melan-A/MART-1_16–40_-FITC (GHGHSYTTAEELAGIGILTVILGVLK-FITC) (SLP_16–40_-FITC) and FITC-Melan-A/MART-1_16–40_ (FITC-GHGHSYTTAEELAGIGILTVILGVL) (FITC-SLP_16–40_), were produced with purity greater than 95%. All the peptides were reconstituted at 10 mM in DMSO.

### Immunofluorescent Staining, Flow Cytometry Analysis and Elisa Assay

CD8 T cell activation by DCs loaded in vitro with SLP_16–40_ or SP_26–35_ peptides was determined by using APC-labeled anti-CD8^+^mAb and PE-labeled anti-IFNγ. Mo-DCs were stained by PKH-67 according to the manufacturer’s recommendations (Sigma) in order to exclude them from the T cell gate.

For intracytoplasmic IFNγ staining, cells were stained at 4°C for 20 minutes, with anti-CD8^+^ Ab. Then cells were fixed 10 minutes at room temperature in PBS 4% paraformaldehyde (Sigma). Anti-IFNγ was added to fixed cells and incubated for 30 minutes at room temperature. Reagent dilutions and washes were done with PBS containing 0.1% BSA and 0.1% saponin (Sigma). After staining, immunofluorescence was analyzed on a FACS calibur (BD Biosciences).

Alternatively, activation of the 10C10 CTL clone was evaluated by determining the IFNγ content in the supernatant in duplicates in a 16-hr CTL assay using a commercially available ELISA kit (BD Biosciences).

### SDS-PAGE and Western Blotting

For the preparation of whole cell lysates, cells were lysed in a buffer containing 50 mM NaCl, 50 mM Tris, 5 mM MgCl2, 1 mM DTT, 10% glycerol and 0.1% NP40. Protein concentrations in lysates were determined using a bicinchoninic acid assay (BCA). Five to twenty µg of whole-cell lysates were separated on a 15% SDS-polyacrylamide gel and transferred onto a PVDF membrane. In some experiments 200 ng of purified 20S proteasome from erythrocytes (Standard Proteasome) or spleen (Standard Proteasome and ImmunoProteasome) were loaded as internal controls. The blots were probed with antibodies to β1i, β5i, Beta1, Beta2, Beta5, Alpha6 (all purchased from Enzo life sciences), β2i (K65/4, laboratory stock), p97/VCP (MA3-004, Dianova), Derlin-1, sec61-a and to β-actin (Santa Cruz Biotechnology) to confirm that equal amounts were present in every lane. Bound antibodies were visualized with ECL chemiluminescence (Roche).

### Antigenic Pulse and Chase, Drug Treatment of DC or SLP_16–40_ Cross-presentation Assays

SLP_16–40_ was pulsed at 10 µM, 37°C for 3 h on DCs in RPMI supplemented with 2% human albumin, 1000 U/mL GM-CSF, 200 U/mL recombinant human IL-4, 1000 U/mL TNFα and 50 µg/mL poly I:C. Following the pulse, 1.10^5^ DC were plated per well in 96-well round-bottom plates, fixed with 0,01% glutaraldehyde containing PBS for 1 min then washed three times in RPMI. DCs were co-cultured with T-cells in the presence of 10 µg/mL Brefeldin A (BFA).

In some assays, DCs were incubated for 30 minutes before and throughout the antigen pulse period with Cytochalasine D (Sigma), ICP_47_ (1–35) (Millegen (Labege, France), *Pseudomonas aeruginosa* Exotoxin A (Sigma), NH_4_Cl (Sigma), or Epoxomicin (Sigma).

1.10^5^ pulsed-DCs were co-cultured for 6 h with the 10C10 clone using 1.10^5^ T-cells per well, in a final volume of 100 µL of RPMI containing 8% human serum and 10 µg/ml brefeldin A (Sigma, St Louis MO, USA). The DC/T cell ratio was 1∶1.

### siRNA Transfection of DC

ON-TARGET plus SMART pool of interfering RNA (Dharmacon) were used to knock down p97/VCP (L-008727-00), sec61A1 (L-021503-00), Derlin-1 (E-010733-00), β1i (L-006023-00), β2i (L-006019-00) and β5i (L-006022-00). ON-TARGET plus nontargeting pool of siRNA with random nucleotides (D-001810-10) was used in each experiment as a negative control. For siRNA transfection, 4.10^7^ cells were resuspended in 100 µl Opti-MEM without phenol red (Invitrogen) and transferred into a 4-mm electroporation cuvette (Biorad) with 1000 nmol siRNA duplex. The electroporator (Genepulser, Biorad) used a square-wave pulse of 500 V for 1 ms. Cells were then immediately transferred into 4 ml of MoDCs medium.

### Confocal Microscopy

The mouse IgG1-mAb used in confocal microscopy were anti-HLA class-I (W6–32, produced in our laboratory), anti-GM130, anti-EEA-1, anti-LAMP-1 and anti-calreticulin (Becton Dickinson), that target respectively HLA-A,B,C and the following subcellular compartments - Golgi apparatus, early endosomes, lysosomes and ER.

DCs were pulsed for 0–48 h with SLP Melan-A_16–40_ FITC (10 µM) in MoDCs maturation medium. Following the pulse, DCs were fixed in a solution of 2% formaldehyde (Sigma) and stained at room temperature for 2 h either by anti-HLA or, after permeabilization in a solution of PBS containing 0.05% triton X100 and 0.05% Tween, by the mouse IgG1-mAb, anti-GM130, anti-EEA-1, anti-LAMP-1 or anti-calreticulin. Cells were washed 3 times in PBS and secondary Alexa-568 anti-mouse IgG1 (Invitrogen) was used as detection reagent. Cells were washed and nuclei were visualized with DRAQ 5 (AXXORA). Isotype control antibodies were used in all confocal microscopy experiments to confirm specificity of antibody staining. Coverslips were mount in prolong gold antifade reagent (Invitrogen) and examined with confocal microscopy. Z-series of multiple images were acquired from DCs representative of most cells in each culture.

Cell fluorescence was visualized by confocal microscopy using a NIKON A1, R, SI instrument with APO VC, X60 NA: 1,4 oil immersion objective. An argon laser at 488 nm and diode laser at 561 nm excited the fluorescence of FITC and Alexa 568 respectively; fluorescence emission was collected respectively at 525/15 for FITC and 590/15 for Alexa-568. All images were acquired at a size of 1024 pixel by 1024 pixel and had a lateral resolution of 0.21 µm by pixel. Z-step numbers and rank were chosen according to Nyquist theorem where Z = 1/3 FWHM (Full Width at Half Maximum): typically 0.25 µm for Z-range.

For analysis, Image J software was used. Colocalization volumes were calculated with the 3D object counter plugin [28].

Quantitation of the colocalization of SLP_16–40_ with the different subcellular compartments (Golgi apparatus, early endosomes, lysosomes or ER) was performed by calculating the Pearson correlation coefficient, Rr, using Volocity software (Version 6.1.1 from PerkinElmer Life Sciences). An Rr value of 1 indicates complete colocalization, an Rr value of 0 indicates no specific colocalization, and an Rr value of −1 indicates a perfect but inverse correlation (exclusion). The green and red fluorescence have been determined by performing thresholding using the 3D object counter plugin default threshold method.

### Statistical Analysis

For the analysis of experimental data, quantitative data was compared by the Mann-Whitney U test (Graph Pad Prism 5).

## Results

### Efficient Cross-presentation of SLP Melan-A_16–40_ FITC Peptides by DC

**Figure 1:**
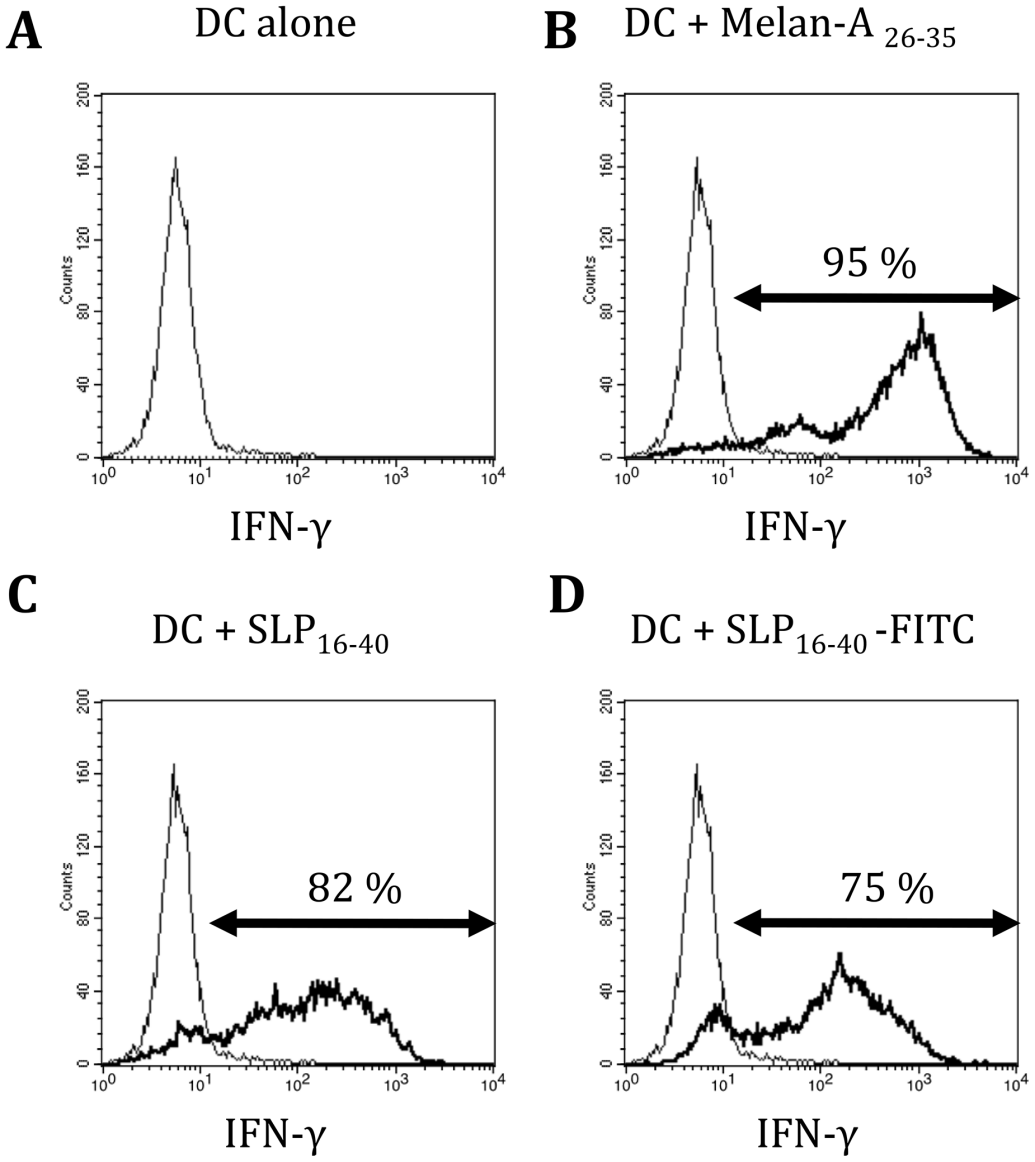
SLP_16__–40_-FITC is cross-presented efficiently by the DC. Flow cytometry of DC activation of the 10C10 CD8+ T-cell clone 10C10. DC were pulsed for 3 h in the presence of short peptide MelanA_26–35_, SLP_16–40_, or SLP_16–40_-FITC before co-culture with the 10C10 clone at a 1∶1 cell ratio. The histogram represents the fluorescence emitted by the 10C10 clone stained with anti-IFN-γ.

In previous studies, others and we showed that the SLP_15–40_ or SLP_16–40_ are cross-presented by DC [25], [29]. To study the cellular mechanisms involved in cross-presentation of the analog long peptide Melan-A_16–40_ A27L, a SLP, which includes the 10-mer Melan-A/Mart-1_26–35_ epitope, was synthesized and coupled with FITC either to the N-terminus (FITC-SLP_16–40_) or to the C-terminus (SLP_16–40_-FITC). Previous results showed that fixed DC pulsed with SLP_16–40_ were unable to stimulate a CTL clone specific for Melan-A/MART-1_26–35_. In order to investigate the impact of FITC addition on cross-presentation, HLA-A2 DCs were incubated with different SLP (SLP_16–40_, FITC-SLP_16–40_, SLP_16–40_-FITC) for 3 hours. Then the 10C10 CTL clone was added for 5 hours. Cross-presentation by DC of SLP_16–40_ was evaluated by the IFNγ response of the T-cell clone. As shown in Figure 1, SLP_16–40_-FITC was cross-presented by DCs as efficiently as its non-labeled analogue. In contrast, FITC-SLP_16–40_ did not induce a response in the 10C10 clone and therefore was not cross-presented (data not shown). We thus used SLP_16–40_-FITC to study the intracellular pathway involved in SLP cross-presentation.

### Visualization of SLP_16–40_-FITC Internalization by DCs

**Figure 2:**
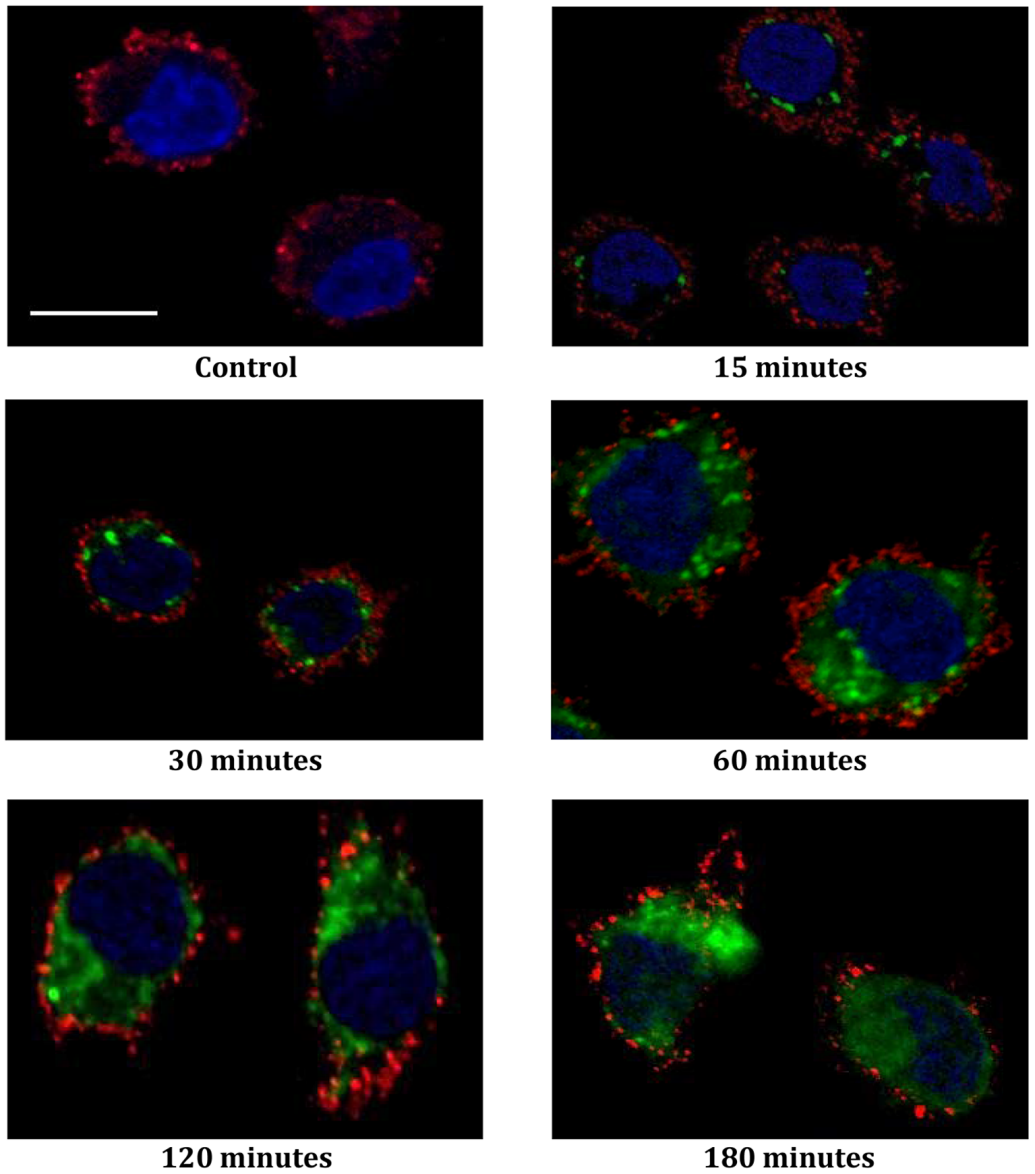
SLP_16__–40_-FITC fluorescence monitored by confocal microscopy. Immunofluorescence microscopy. Kinetics of internalization of SLP_16–40_-FITC (green) by DCs. DCs were stained at the cell membrane with antibody w6–32 (red) and nuclei were counterstained with DRAQ 5 (blue). All image represent one single optical section. Step size of 0,5 µm thick. Original magnification, X60. Data are representative of two independent experiments. Scale bar represent a distance of 5 µm.

In order to visualize the routing of SLP_16–40_, DCs were pulsed with SLP_16–40_-FITC for different times, then fixed and stained with mAbs specific for MHC class-I molecules (W6–32) and a DNA dye specific for the nucleus (Draq5). As shown in Figure 2, SLP_16–40_-FITC internalization was detectable in DC as early as 15 minutes after the pulse by monitoring the FITC tag. Using Immunofluorescence confocal microscopy, we observed that DCs exhibit morphology typical of intermediate-stage DCs, with most cells having a rounded shape (Fig. 2). Thereafter, we visualized a progressive internalization and accumulation of SLP_16–40_-FITC in DCs between 15 min and 180 min of pulse-chase. These results demonstrate that SLP_16–40_ labeled with FITC is efficiently detectable in DCs by confocal microscopy and the signal persists during its intracellular routing.

### Specific Routing of SLP_16–40_ toward Early Endosomes and Lysosomes

**Figure 3:**
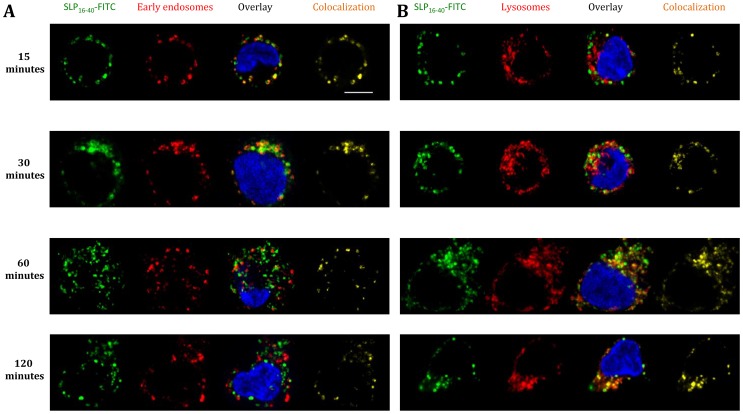
Study of SLP_16__–40_-FITC colocalization in DCs : colocalization in early endosomes and lysosomes. (A) Immunofluorescence microscopy. Kinetics of internalization of DCs incubated with SLP_16–40_-FITC (green), 15, 30, 60 or 120 minutes after pulse. DCs are stained at the early endosomes with antibody anti-EEA-1 (red) and the nuclei are counterstained with DRAQ 5 (blue). All image represent one single optical section. Step size of 0,5 µm thick. Original magnification, X60. Single scans are representative for multiple cells analysed in at least 2 experiments. (B) Immunofluorescence microscopy. Kinetics of internalization of DCs incubated with SLP_16–40_-FITC (green), 15, 30, 60 or 120 minutes after pulse. DCs are stained at the lysosomes with antibody LAMP-1 (red) and the nuclei are counterstained with DRAQ 5 (blue). All image represent one single optical section. Step size of 0,5 µm thick. Original magnification, X60. Single scans are representative for multiple cells analysed in at least 2 experiments. Scale bar represent a distance of 5 µm.

**Figure 4:**
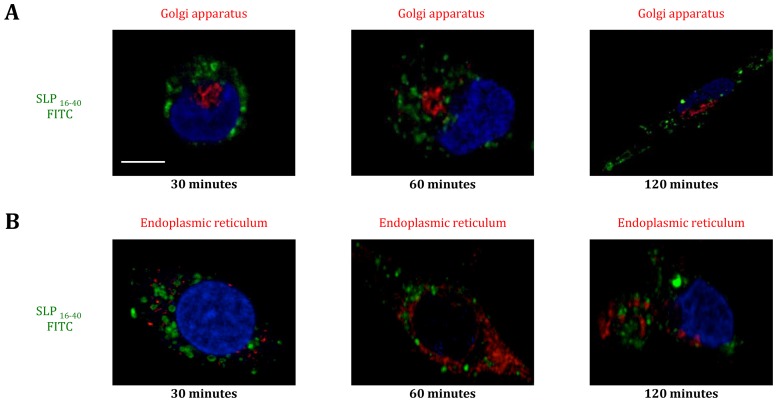
Study of SLP_16__–40_-FITC colocalization in DCs: no colocalization in Golgi and ER. (A) Immunofluorescence microscopy. Kinetics of internalization of DCs incubated with SLP_16–40_-FITC (green), 30, 60 or 120 minutes after pulse. DCs are stained at the Golgi with antibody anti-GM130 (red) and the nuclei are counterstained with DRAQ 5 (blue). All image represent one single optical section. Step size of 0,5 µm thick. Original magnification, X60. Data are representative of two independent experiments. (B) Immunofluorescence microscopy. Kinetics of internalization of DCs incubated with SLP_16–40_-FITC (green), 30, 60 or 120 minutes after pulse. DCs are stained at the ER with antibody anti-calreticulin (red) and the nuclei are counterstained with DRAQ 5 (blue). All image represent one single optical section. Step size of 0,5 µm thick. Original magnification, X60. Data are representative of two independent experiments. Scale bar represent a distance of 5 µm.

To define the intracellular routing, we incubated DCs for different periods of times (0 to 120 minutes) with SLP_16–40_-FITC and then stained DCs intracellularly with antibody to early endosomes (anti-EEA-1, Fig. 3A) or lysosomes (anti-LAMP-1, Fig. 3B). Between 15 and 60 minutes after SLP_16–40_-FITC incubation, DCs showed strong colocalization between SLP_16–40_ and early endosomes (Fig. 3A). After this period, no colocalization could be observed with this compartment (Fig. 3A). Concerning lysosomes, colocalization started at 30 minutes until 120 minutes (Fig. 3B), thus, we propose that SLP_16–40_ could be redirected from endosomes to lysosomes. No colocalization was detectable between SLP_16–40_-FITC and the Golgi apparatus (anti-GM130) or the ER (anti-calreticulin) (Fig. 4). Altogether, our results indicate that SLP_16–40_ was internalized by DCs in early endosomes and later reached the lysosomes.

### Kinetics of Internalization of SLP_16–40_ in DCs

**Figure 5:**
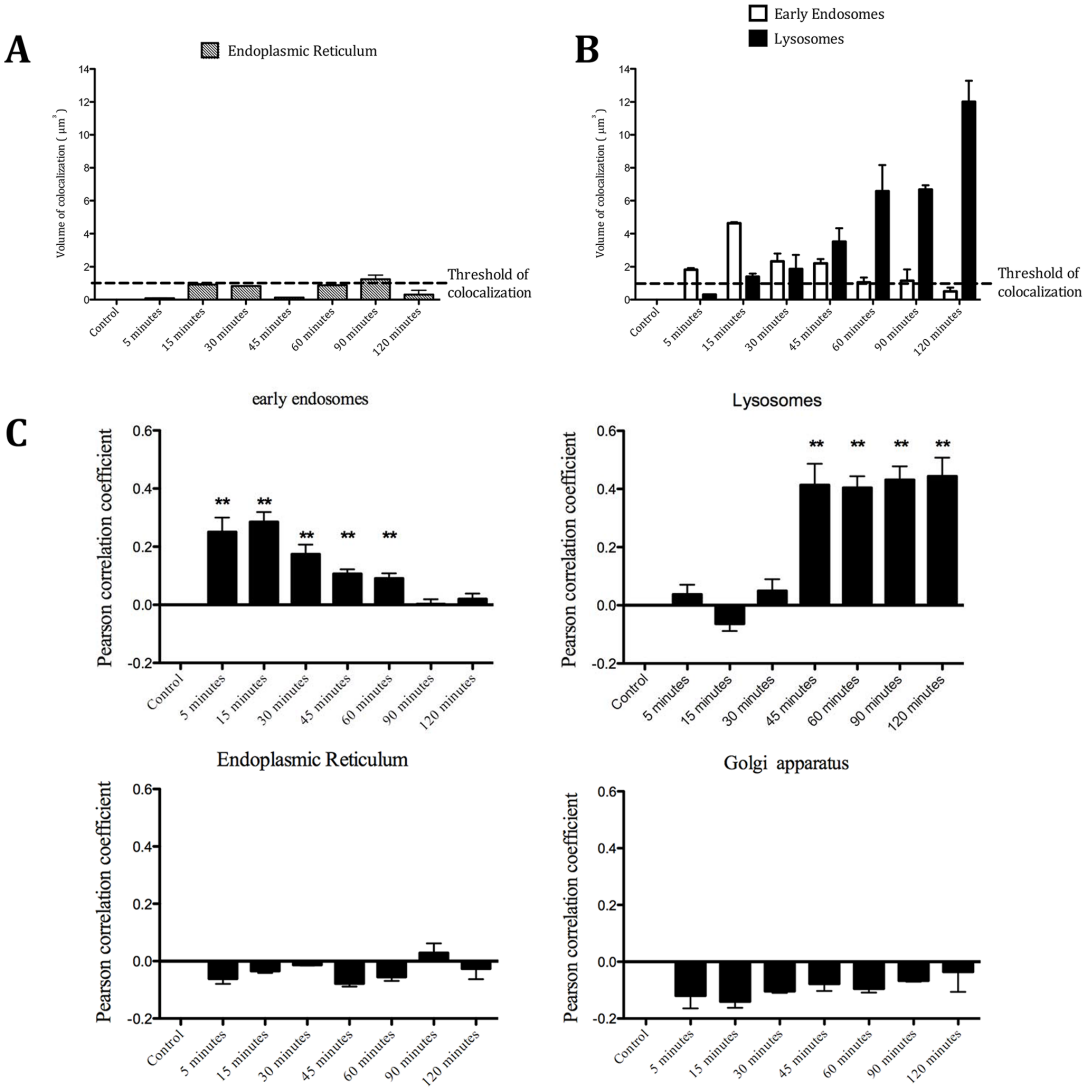
Kinetics of internalization of SLP_16__–40_ in DCs. (A) Volume measurement of green fluorescence (SLP_16–40_-FITC) with red fluorescence (ER) colocalization at each time point. For each point, average volume was determined on five different cells of two independent experiments. (B) Volume measurement of green fluorescence (SLP_16–40_-FITC) with red fluorescence colocalization (early endosomes or lysosomes) at each time point. Representations of colocalization in early endosomes are in white bars and colocalization in lysosomes are in black bars. For each point and each compartiment, average volume was determined in five different cells from two independent experiments. (C) The colocalization of SLP_16–40_-FITC immunofluorescence with intracellular compartments (early endosomes, lysosomes, ER and Golgi apparatus) was quantified by measuring the Pearson correlation coefficient (Rr) with Velocity software. A Pearson correlation of 1 indicates complete colocalization, a value of 0 indicates no specific colocalization and a value of −1 indicates a perfect but inverse correlation (exclusion). Measurements of the Pearson correlation coefficient indicate a reasonable degree of partial colocalization of SLP_16–40_ with early endosomes between 5 and 60 minutes and with lysosomes between 45 and 120 minutes. The Pearson correlation coefficient of SLP_16–40_ with ER and Golgi apparatus lysosomes indicates no specific colocalization. The Pearson correlation coefficient was measured with n = 5 cells. Statistical significance of colocalization was compared to the null hypothesis of no specific colocalization (Pearson correlation coefficient value of 0).

To address more precisely the localization of SLP_16–40_ in DCs, we analyzed confocal microscopy images of each cell with the Image J 3D cell counter plugin. This application allows the measurement of the colocalization volume defined by the overlap of the green fluorescent object (SLP_16–40_) with the red fluorescent object (intracellular compartment) in the whole cell. To this end, we defined a threshold of no colocalization with the endoplamic reticulum staining (Fig. 5A).

We evaluated the colocalization volume for early endosome and lysosome (Fig. 5B). Between 5 and 30 minutes, SLP-FITC_16–40_ colocalization was high in early endosomes and weak in lysosomes. Between 30 and 120 minutes, the trend reversed and the colocalization seemed to increase in lysosomes. Moreover, we observed that SLP_16–40_-FITC accumulated in lysosomes from 30 to 120 minutes post-pulse.

Quantitation of SLP_16–40_-FITC colocalized with early endosomes, lysosomes, ER and Golgi apparatus was measured by the Pearson correlation coefficient (Rr value). Rr values indicate specific partial colocalization of SLP_16–40_ with early endosomes between 5 and 60 minutes and with lysosomes between 45 and 120 minutes (Figure 5C). This demonstrates that the volumes of detected colocalization represent the specific colocalization between the SLP_16–40_ and this two subcellular compartments rather then a juxtaposition of fluorescence. On the other hand, Rr values indicate no specific colocalization of SLP_16–40_ with the ER or Golgi apparatus, in accordance with microscopy images displayed.

Overall, these data suggest that SLP_16–40_ is rapidly internalized by DCs in early endosomes, and then redirected to the lysosomes where it accumulates.

### Proteasomal Processing of SLP_16–40_

**Figure 6:**
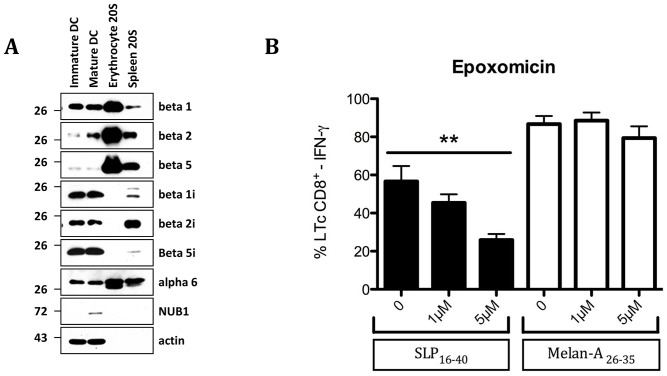
Immature and mature DC express mixed-type proteasomes. (A) Untreated day 5-immature DC and DC treated with LPS (1 µg/ml) for 24 hours were analysed for their proteasome content by western-blotting using antibodies against β1, β2, β5, β1i, β2i, β5i, as indicated. Purified 26 proteasomes (250 ng) from erythrocytes (standard proteasome) and spleen (mixture of standard and immunoproteasome) were used as sources to ensure antibody specificity. To control for equal loading, proteins were subjected to western blotting using the anti-β-actin antibody. (B) Flow cytometry of DC recognition by CD8^+^ T-cell clone 10C10. DCs were treated with Epoxomicin (1 and 5 µM) for 30 min, then DCs were pulsed for 3 h in the presence of short peptide MelanA_26–35_, or SLP_16–40_, and inhibitor before co-culture with the 10C10 clone at a 1∶1 cell ratio. Data are representative of at least three independent experiments. Statistical analysis was performed using non-parametric Mann-Whitney test and values in the presence of inhibitor were significantly different (p<0,04).

Various proteases may be involved in the processing of SLP_16–40_. In DCs, the proteasome is involved in generation of many MHC-I epitopes from endogenous proteins (endogenous pathway) but also from exogenous proteins during cross-presentation [30]. The standard proteasome and the immunoproteasome are multimeric complexes characterized by the catalytic subunits (β1, β2, β5) and (β1i, β2i, β5i) respectively. Apart from the standard proteasome, there are at least three subtypes of immunoproteasome: two intermediate proteasomes (β5i and β1i, β5i) and the classical immunoproteasome [31]. We found that DCs used in the present study essentially expressed the immunoproteasome or intermediate proteasomes because few subunits of standard proteasome were detectable (Fig. 6A).

To assess whether early cross presentation of SLP_16–40_ by DCs requires proteasome degradation for presentation, we used epoxomicin. This drug is a highly specific, and irreversible inhibitor of the chymotrypsin-like (CT-L), trypsin-like (T-L), and peptidyl-glutamyl peptide hydrolyzing (PGPH) activities of the proteasome which modifies the proteasomal catalytic subunits β5i, β2i, β5 and β2 [32] DCs were preincubated with different doses of epoxomicin (1 and 5 µM) then incubated with SLP_16–40_. Epoxomicin strongly inhibits the DC cross-presentation capacity in a dose dependent manner (Fig. 6B) but had no effect on the exogenous presentation of synthetic 10-mer epitope Melan-A/Mart-1_26–35_.

**Figure 7:**
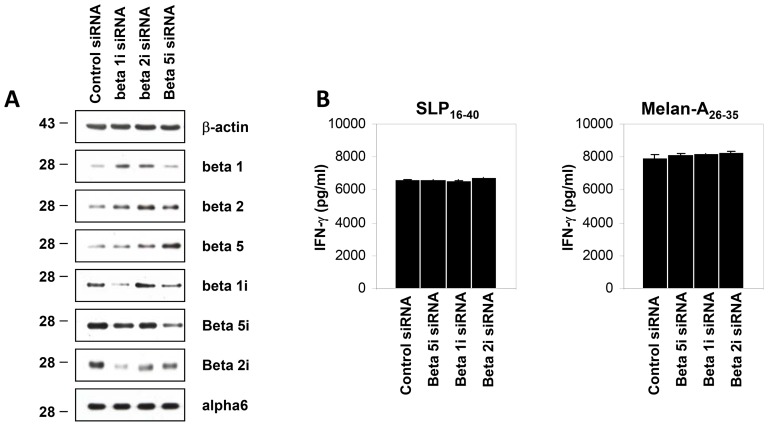
Effect of siRNA depletion of each of the inducible proteasome subunit β1i, β2i, β5i on the DC-mediated cross-presentation of the SLP 16–40. (A) DC were transfected with 1 µM of control siRNA or β1i, β2i, β5i -targeting siRNA for 72 hours. The knockdown of the above-stated inducible subunits as well as its impact on the steady-state level of each of the standard proteasome subunits (β1, β2, β5) was analysed by western-blotting using specific antibodies, as indicated. Antibody against b-actin was used to ensure an equal protein loading. (B) IFN-g production of the LT CD8+10C10 responded to β1i, β2i, β5i -depleted DC pulsed with either SLP16–40 or Melan- A26–35. All data are shown as means +/− SD and are representative of three independent experiments.

To better understand the role of immunoproteasomes in this process, DC with a knockdown of any one of the three inducible subunits β1i, β2i or β5i were fed with SLP_16–40_ for 3 h prior to a 16-hour CTL assay in the presence of the 10C10 clone. As shown in Fig. 7A, gene silencing via specific siRNA was efficiently achieved at the protein level for each of the inducible subunits within 72 h of transfection. Of note, β1i depletion was accompanied by a slight but significant decreased expression of both β1i and β2i. This result is in agreement with previous studies showing that β5i incorporation is a prerequisite for β1i incorporation which, itself, is required for maintaining normal levels of β2i [33]. In addition, knockdown of β5i resulted in increased expression of its standard subunit counterpart β5 but also, albeit to a smaller extent, of β2. Similarly, targeted disruption of either β1i or β2i caused an up-regulation of both of the standard subunits β1 and β2.

However, and in spite of the up-regulation of at least any of two out of three standard subunits in DC treated with a knockdown of anyone of the three inducible subunits, no major change in SLP_16–40_ cross-presentation could be observed (Fig. 7B). These results indicate that the generation of the Melan-A_26–35_ peptide from SLP_16–40_ in DC for cross-presentation occurs independently of the proteasomal subunit composition.

### Cross-presentation of SLP_16–40_ is Dependent on Functional TAP Transport

**Figure 8:**
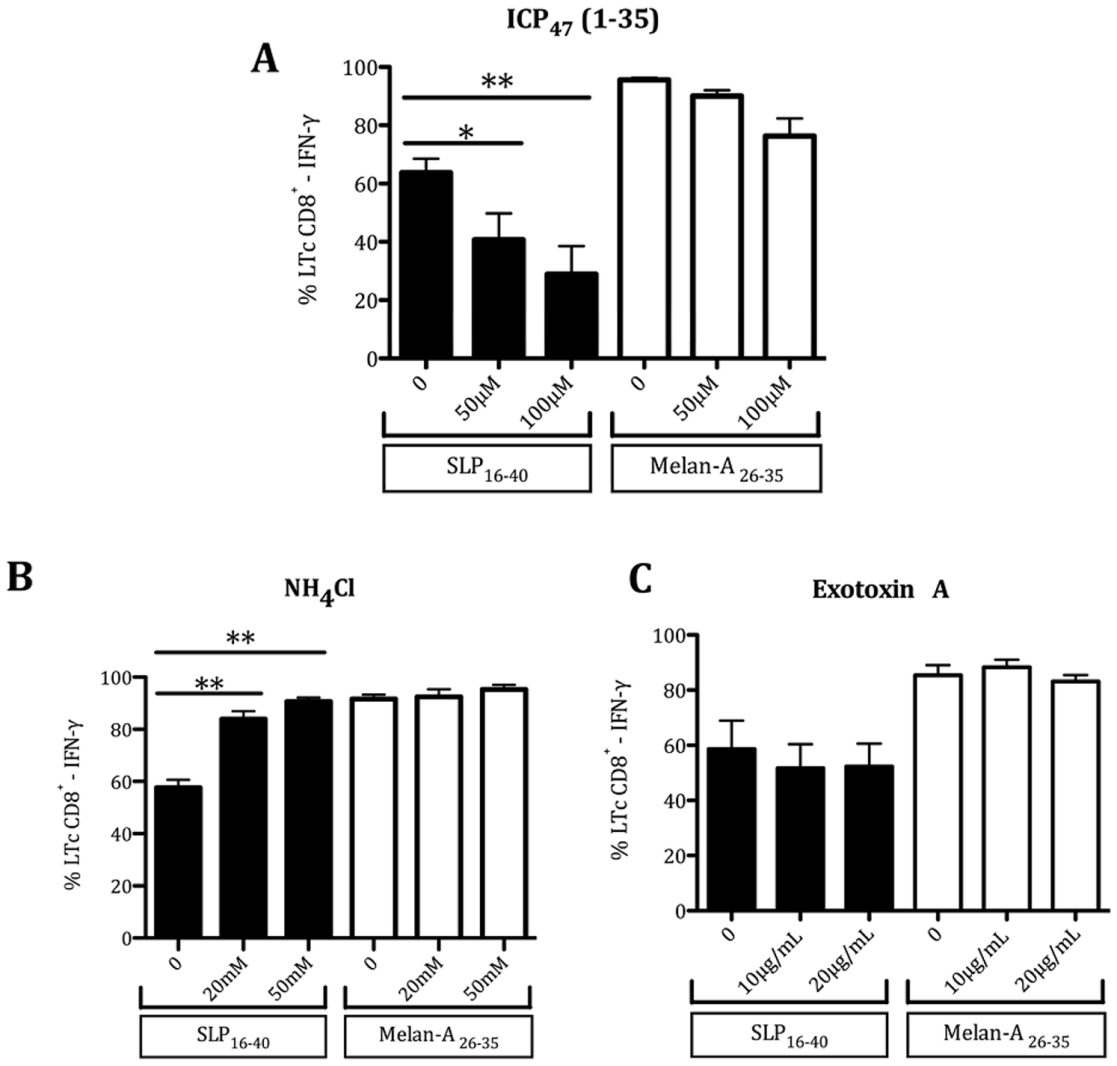
Effects of different inhibitors on SLP_16__–40_ cross-presentation. (A) TAP transport: Flow cytometry of DC recognition by LT CD8^+^10C10. DCs were treated with inhibitor, ICP47 (50 and 100 µM) for 30 min, then DCs were pulsed for 3 h with the short peptide Melan-A_26–35_, or SLP_16–40_, and in the presence of the inhibitor before co-culture with the LT CD8^+^10C10 at a 1∶1 cell ratio. Data are representative of at least three independent experiments. Statistical analysis was performed using non-parametric Mann-Whitney’s test and values in the presence of inhibitor were significantly different (p<0.04). The molecular mechanisms involved in cross-presentation of SLP_16–40_. Flow cytometry of DC recognition by LT CD8^+^10C10. DCs were treated with inhibitor, (B) NH_4_Cl (20 mM and 50 mM) or (C) ExoA (10 and 20 µg/mL) for 30 min, then DCs were pulsed for 3 h in the presence of short peptide Melan-A_26–35_, or SLP_16–40_, and inhibitor before co-culture with the LT CD8^+^10C10 at a 1∶1 cell ratio. Data are representative of at least three independent experiments. Statistical analysis was performed using non-parametric Mann-Whitney’s test and values in the presence of inhibitor were significantly different (p<0,04).

In the cytosol, the antigens degraded into peptides are transported into the ER or ER phagosome-like compartments via the transporter associated with antigen processing (TAP) for loading onto MHC class I molecules. To investigate whether TAP was involved in the cross-presentation of SLP_16–40_, we used a synthetic peptide corresponding to the N-terminal 35 amino acid residues (ICP_47_ (1–35)), that can reach the cytosol and block TAP only in cells of DC lineage [34]. DCs were preincubated with ICP_47_ (1–35) and cocultured for 3 hours with SLP_16–40._ We showed that ICP_47_ (1–35) decreased cross-presentation of SLP_16–40_ by DCs (Fig. 8A) with a slight but no statistically significant, inhibitory effect on the exogenous presentation of synthetic 9-mer epitope Melan-A/Mart-1_26–35_. These findings suggest that TAP play a role in cross-presentation of peptide from SLP_16–40_ in cytosol.

### SLP_16–40_ Cross-presentation Depends on Early Endosomes and Retrotranslocation Machinery

To define more precisely the compartments involved in SLP_16–40_ cross-presentation by DCs, we incubated DCs for 3 hours with SLP_16–40_ in the presence or absence of various inhibitors. First, to elucidate the potential lysosomal involvement in cross-presentation, we used NH_4_Cl, an inhibitor of lysosome acidification and maturation of early endosomes into lysosomes [35]. As shown in Fig. 8B, NH_4_Cl treatment increased antigen cross-presentation of SLP_16–40_ without modifying the exogenous presentation of synthetic 10-mer epitope Melan-A/Mart-1_26–35_. The increased cross-presentation is likely due to an accumulation of SLP_16–40_ in early endosomes due to the inhibition by NH_4_Cl. These results suggest that lysosomes do not participate in SLP_16–40_ cross presentation and points out a role for early endosomes in cross-presentation.

Following its internalization in early endosomes, translocation of SLP_16–40_ to the cytosol is required for subsequent degradation by cytosolic proteases and/or the proteasome [8]. It has been shown that such transport across the endosomal membrane can be achieved via the ER-associated degradation pathway (ERAD) [34], [36]. Indeed, the ERAD machinery normally and usually allows the retro-translocation of misfolded proteins from the ER back to the cytosol, a process that is thought to be mediated by the channels sec61 and/or Derlin-1 as well as the AAA-ATPase p97/VCP [37], [38]. Because sec61 is expressed on early endosomes [39] and might play a role in antigen translocation [40], [41], we next sought to determine the contribution of this protein to SLP_16–40_ cross-presentation using the sec61 inhibitor Exotoxin A (Exo A). As shown in Fig. 8C, the use of this inhibitor tended to decrease slightly the cross-presentation of SLP_16–40_ but this decrease was not significant.

**Figure 9:**
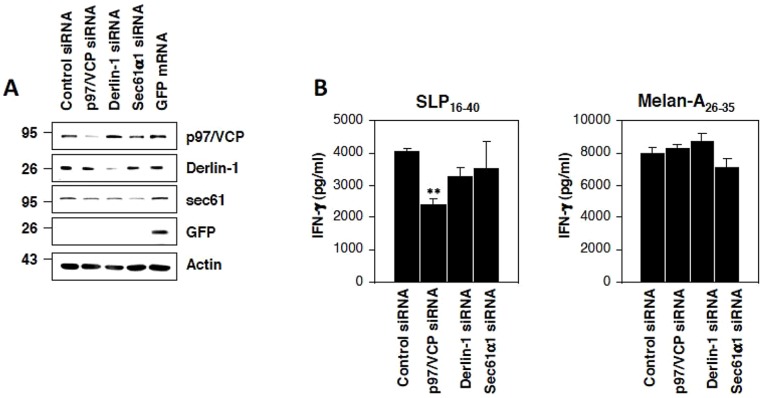
Gene silencing of the ERAD-related component p97/VCP substantially impairs cross-presentation of the SLP16–40. (A) Day5-immature HLA-A2+ DC were electroporated with 1 µM of either control siRNA or siRNA against p97/VCP, Derlin-1 or Sec61a1, as indicated. The steady-state protein level of each of the targeted gene was determined by western-blot analysis 72 hours later. To control for equal loading, samples were subjected to western blotting using the anti-b-actin antibody. (B) The Melan-A26–35 CTL response against siRNAtreated DC loaded with either 10 µM of SLP16–40 or 1 µM of Melan-A26–35 (as a positive control) was examined using an IFN-γ ELISA. All data are shown as means +/− SD and are representative of three independent experiments. **p<0.01 (t-Test).

In an attempt to generate a more specific and reliable blockade of the ERAD pathway in DC, we generated knockdown DC for each of the three above-mentioned ERAD factors (i.e., p97/VCP, Derlin-1 and sec61a1). To this end, DC were treated with siRNA targeting p97/VCP, Derlin-1 or sec61a1 for three days followed by a 3-hour pulse of either SLP_16–40_ or Melan-A_26–35_ prior to a 16-hour CTL recognition assay in the presence of the 10C10 clone. As shown in Fig. 9A, treatment of DC with any of the above-mentioned siRNA resulted in significant diminished expression of the corresponding protein within 72 h of transfection. Interestingly, from the three ERAD components tested, only p97/VCP was found to significantly impair SLP_16–40_ cross-presentation (Fig. 9B). Taken together, these data show that the SLP_16–40_ is internalized in early endosomes where it is translocated into the cytoplasm by a retro-translocation complex involving p97/VCP.

## Discussion

The aim of this study was to characterize the SLP cross-presentation pathway in DCs. We selected for the present *in vitro* study a 25-mer sequence of the Melan-A/MART-1 melanoma associated antigen bearing an anchor optimized analog of the immunodominant CD8^+^ epitope (epitope of Melan-A: Melan-A_26–35_ A27L on HLA-A2) [22].

**Figure 10:**
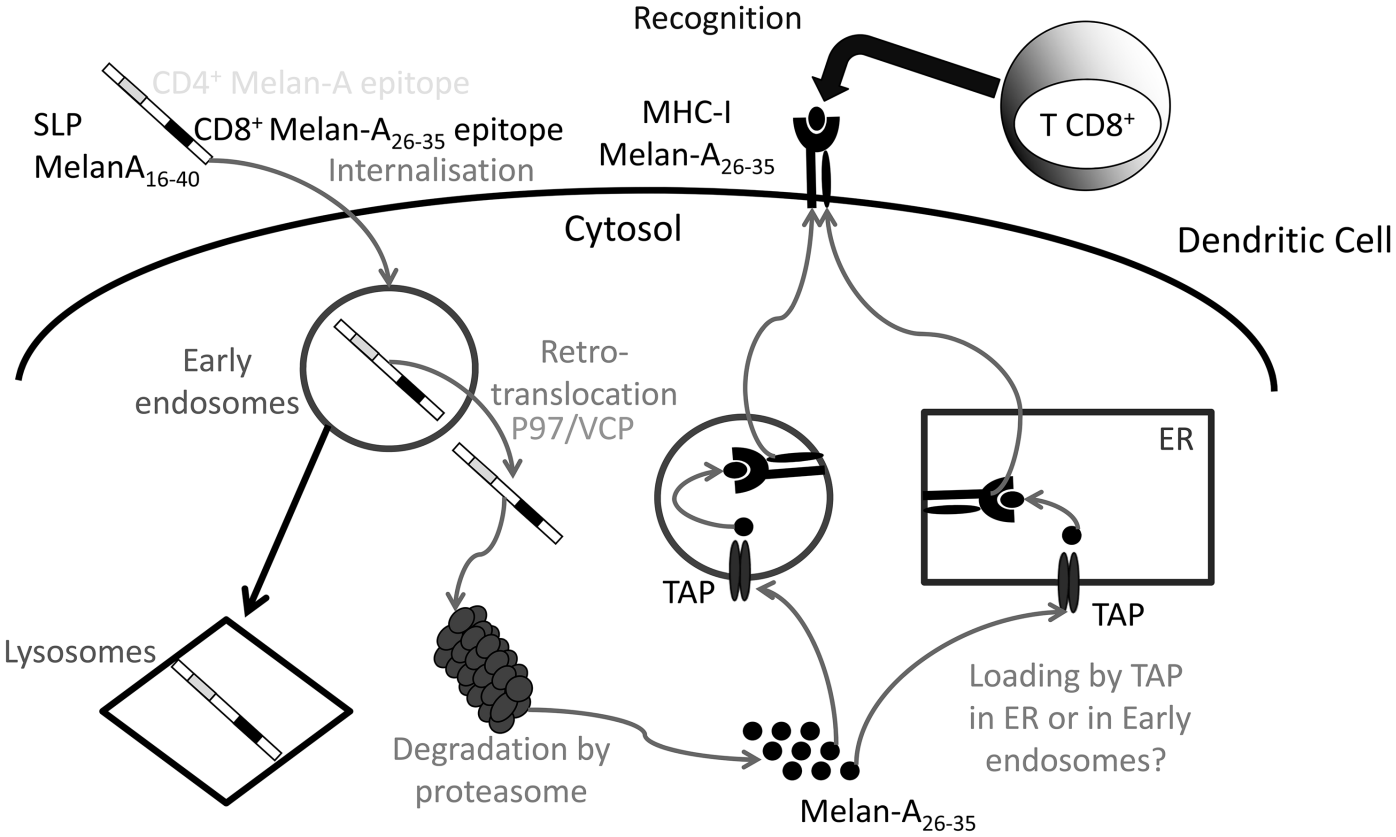
Putative cross-presentation mechanisms of SLP Melan-A_16__–40_.

Our principal results concerning SLP_16–40_ cross-presentation are summarized in Figure 10: SLP_16–40_ is internalized by DCs into early endosomes. Part of it is directed into the lysosomal compartment. The remainder is exported into the cytoplasm by a retrotranslocation complex involving p97/VCP. In the cytoplasm, SLP_16–40_ is processed by the proteasome and the resulting Melan-A peptide fragment is reimported by TAP into the endosomal or ER compartment for loading onto MHC class I molecules. Inhibition of lysosome acidification increased Melan-A_26–35_ epitope generation, in contrast to the effect previously documented by Fonteneau et al., for a different antigen: influenza matrix protein [42]. This pathway is a mix between the “endocytic” and “cytosolic” tracks proposed by Segura and Villadongos [30] and confirm the processing of SLP-OVA_24aa_ recently published for murine DCs [43].

Here, we demonstrated, by using inhibitors and confocal microscopy that SLP_16–40_ is endocytosed by DCs and routed to early endosomes. The results showed that SLP_16–40_ was internalized very quickly after DC contact (15 to 30 minutes). Later on, specific colocalization of peptide with endosomes decreased and then disappeared from 60 minutes onwards, together with the appearance of SLP_16–40_ colocalization with lysosomes. The decreasing colocalization between early endosomes and SLP_16–40_ over time suggests an arrest of the endocytic process. One hypothesis is that DCs rapidly internalized a large quantity of SLP reaching a maximum and perhaps preventing supplemental endocytosis during the 3 h incubation with this 25-mer.

We observed a temporal correlation of colocalization between early endosomes and lysosomes (Fig. 4). This suggests that, after internalization, SLP is routed into early endosomes then redirected to and accumulated in lysosomes. This routing could be explained by maturation of DCs: early endosomes becomes late endosomes then lysosomes [44]. The colocalization and accumulation of SLP_16–40_ in lysosomes after 60 minutes may represent an antigen storage compartment that could facilitate long-lasting cross-presentation of SLP_16–40_ by DCs – the “lysosome-like organelles” proposed by other groups. [12]. Indeed, Amigorena’s group has showed that DCs pulsed for 3 hours with the SLP Melan-A_15–40_ allows efficient, long-lasting, cross-presentation of Melan-A/MART-1 tumor antigen on MHC class I molecules from an intracellular antigen storage compartment [29]. Furthermore, similar long-lasting cross-presentation by DCs was also observed for IgG-OVA complexes. In this case, lysosome like organelles were shown to be the intracellular storage compartment allowing long-term presentation of MHC class I/peptide complexes [12]. Otherwise, lysosomes are involved in generation of CD4^+^ T-cell epitopes to produce the MHC-II/peptide complex at the cell surface for activation of CD4^+^ T lymphocytes. Activated CD4^+^ T cells can promote inflammation, cooperate in the induction of CD8^+^ T effectors and memory cells, and provide help for B cells to produce anti-tumor antibodies. As the SLP_16–40_ contains potential CD4^+^ T-cell epitopes, SLP storage in lysosomes could also permit the activation of CD4^+^ T cell responses.

By using cytochalasin D that inhibits actin polymerization, we prevented cross-presentation, suggesting that internalization and possibly processing of SLP_16–40_ require cytoskeletal actin rearrangement (data not shown). Receptor mediated endocytosis and macropinocytosis are efficient mechanisms that can guide exogenous antigens into the MHC class I and II presentation pathway in DCs [9] but this remains to be elucidated. As proposed by Quakkelaar and Melief, it could be interesting to experiment the coupling of SLP to adjuvants like TLR ligands or receptor specific ligands in order to reinforce internalization, cross-presentation and the induction of specific T cell responses by SLP [45].

Although various mechanisms have been proposed to explain cross-presentation, the route used by internalized antigens to gain access to the cytosol for proteasomal degradation remains elusive. Over the past few years, an increasing number of a studies point to a critical role of the ER-associated degradation pathway (ERAD) in this process [34], [41], [46]. The ERAD pathway is a conserved multistep process that normally ensures the transfer of misfolded proteins from the ER back to the cytoplasm for subsequent destruction by the 26S proteasome (an event also termed “retro-translocation”). To date, there is disagreement in the field with respect to identity and/or nature of the retro-translocation channel with two different protein complexes being put forward as possible candidates to fulfill this task, namely sec61 and Derlin-1 [47]. Irrespective of this concern, it now well established that all ERAD pathways converge downstream of the retro-translocation channel at p97/VCP, an AAA-ATPase responsible for “pulling” the proteins that have successfully crossed the ER membrane. Our data fully support a role for ERAD in the transport of the SLP_16–40_ into the cytosol, as shown by the decreased SLP_16–40_ cross-presentation observed in p97/VCP-depleted DCs (Fig. 6E).

However, in our hands, neither sec61 nor Derlin-1 gene silencing could significantly alter the SLP_16–40_ cross-presentation by DC. These findings are interesting and raise the possibility of the existence of another yet unidentified channel implicated in this process. Yet, we cannot rule out a participation of Derlin-1 and/or sec61 in other DC-based cross-presentation systems when other antigen sources are applied such as full-length proteins or cell-associated antigens including apoptotic or necrotic cells. Of note, the extraction of proteins from the ER by p97/VCP requires substrate poly-ubiquitylation. Since our SLP_16–40_ is a lysine-free peptide, it is conceivable that the exclusive acceptor site for poly-ubiquitylation may be represented by its N-terminus. Importantly, this implies that any blockade of the N-terminus of the SLP_16–40_ would prevent its cross-presentation by DC. This assumption is supported by the observation that the N-terminally FITC-modified SLP_16–40_ does not lead to the generation of the Melan-A_26–35_ peptide in DC (data not shown).

In DCs, two proteasomes exist: the standard proteasome that contains the active subunits β1, β2 and β5; and the immunoproteasome, which differs only in three active subunits (the immunosubunits β1i, β2i and β5i). Recently, the existence of additional forms of proteasomes, bearing a mixed assortment of standard and inducible catalytic subunits has been identified, which contains only one (β5i) or two (β1i and β5i) of the three inducible catalytic subunits of the immunoproteasome [31]. We were able to confirm the role of proteasomes in SLP_16–40_ cross-presentation by DCs, as shown by the reduced CTL recognition observed with DC treated with epoxomicin (Fig. 5B). This mechanism is likely to involve the participation of the immunoproteasome or intermediate proteasomes, since the standard proteasome is thought to represent less than 8% of total proteasome content in mature or immature DCs [31]. This notion is further reinforced by the fact that siRNA-mediated depletion of anyone of the three inducible subunits in DC failed to influence the SLP_16–40_ cross-presentation (Fig. 5D). Indeed, because the down-regulation of any of the three inducible subunits is accompanied by the reciprocal up-regulation of at least two standard subunits (Fig. 5C), our data tend to imply that standard, mixed-type and immunoproteasomes are equivalent in the generation of the Melan-A_26–35_ peptide from the SLP_16–40_.

This result seems to contradict previous results in which the antigenic peptide Melan-A_26–35_ was generated by the standard proteasome but not by the immunoproteasome [48], [49]. This discrepancy could be explained by the fact that we used a cellular model with DCs, which could represent a more complex system than purified proteasome or immunoproteasome. Many cytosolic proteases are present in DCs and using a cellular model, Melief’s group has recently described the role of a cytosolic peptidase (thimet oligopeptidase) in the generation of the Melan-A/MART-1 epitope [50]. Also we could not exclude the role of cytokines in the modulation of proteasome activity in DCs [51]. Finally in the present work we evaluated the processing of Melan-A epitope via the cross-presentation pathway. Others and we have already described the cross-presentation of Melan-A peptide by DCs [25], [29], [51]. This result may reflect that cross-presentation process could have some differences in the generation of the Melan-A peptide in comparison with the endogenous pathway.

We have demonstrated the necessity of TAP for the cross-presentation of SLP_16–40_. The TAP transporter is expressed both in early endosomes and the ER. The use of US6 or US6 chemically linked to transferrin [10] could help to discriminate between these two potential locations.

Altogether, many vaccination strategies have been used to enhance antitumor responses by exploiting the cross-presentation capacities of DCs. Recently a very encouraging clinical trial for patients with SLP vaccination was published [18]. This work underlines the therapeutic potential of SLP vaccination, which is characterized by the following advantages: easy manufacturing and easy immune monitoring as SLP contain few T cell epitope. Understanding SLP cross-presentation by DCs should contribute to the development and optimization of this immunotherapy technology.
